# Lack of Association between rs2067474 Polymorphism in Histamine Receptor H_2_ Gene and Breast Cancer in Chinese Han Population

**DOI:** 10.1155/2015/545292

**Published:** 2015-04-02

**Authors:** Wen-Ke Cai, Jia-Bin Zhang, Niu-Min Wang, Ying-Lin Wang, Can-Hu Zhao, Jun Lu, Gong-Hao He

**Affiliations:** ^1^Department of Cardio-Thoracic Surgery, Kunming General Hospital of Chengdu Military Region, Kunming 650032, China; ^2^Department of Pharmacy, Kunming General Hospital of Chengdu Military Region, Kunming 650032, China; ^3^Hepatobiliary Surgery Center, 302 Hospital, Beijing 100039, China; ^4^Department of Pharmacy, The First Affiliated Hospital of Medical College, Xi'an Jiaotong University, Xi'an 710061, China; ^5^Department of Anesthesiology, Affiliated Haikou Hospital, Xiangya School of Medicine, Central South University, Haikou 570208, China

## Abstract

Histamine H_2_ receptor (HRH2) was previously suggested to affect the proliferation of breast cancer cells and disease-free survival of breast cancer patients. Furthermore, a common polymorphism, rs2067474, was identified in an enhancer element of the *HRH2* gene promoter and was reported to be associated with various diseases including cancer. However, the relationship between this polymorphism and breast cancer risk and malignant degree remains unclear. The aim of this study was to clarify the clinical association of rs2067474 polymorphism with breast cancer. A total of 201 unrelated Chinese Han breast cancer patients and 238 ethnicity-matched health controls were recruited and rs2067474 polymorphism was genotyped. Logistic regression analyses were performed to calculate the odds ratios (ORs) as a measure of association of genotype with breast cancer according to 3 genetic models (dominant, recessive, and additive). Although the percentage of hormone receptor negative cases tended to be higher in AA genotypes, we did not find any significant associations of rs2067474 polymorphism with breast cancer risk or with related clinicopathological parameters in the present study, which indicates that rs2067474 polymorphism of* HRH2* gene might not be a risk factor in the development of breast cancer in Chinese Han population.

## 1. Introduction

Histamine H_2_ receptor (HRH2) is a kind of Gs protein-coupled receptor, whose activation stimulates adenylyl cyclase and increases intracellular cAMP [1]. This type of histamine receptors was traditionally believed to be mainly expressed in upper gastrointestinal tract, heart, and central nervous system as well and mediated corresponding pathophysiological roles such as gastric muscular atrophy [2], ischemia-induced arrhythmia [3], and schizophrenia [4]. Besides the abovementioned classical places, HRH2 has long been recognized to be expressed in human mammary gland and breast cancer cells [5]. Furthermore, activation of HRH2 in breast cancer cells was reported to increase tumor proliferation [6, 7] and blocking HRH2 was suggested to improve disease-free survival in breast cancer patients [8]. Additionally, HRH2 expressed in other cells (e.g., fibroblasts) was also reported to mediate important effects on the epithelial-to-mesenchymal transition progress of breast cancer cells [9]. These findings strongly indicate that differential expression and function of HRH2 might be a risk factor of breast cancer.

Human HRH2 protein is encoded by* HRH2* gene, which is located in chromosome 5q35.2. Within* HRH2* gene, one single nucleotide polymorphism (SNP), rs2067474, has gained great attention from investigators today. This SNP results in a G1018A transition located in an enhancer element of* HRH2* gene promoter with a relevant allele frequency in diverse human populations according to previous reports [10] and also HapMap data (http://www.hapmap.org/). It was found that rs2067474 was significantly associated with risk of various diseases including cancer [2, 11–13] and presumed to induce changes in the expression of receptors [14]. Based on the notion that genetic variants (including histamine-related gene polymorphisms) contributed greatly to the development of breast cancer and also to the response to specific treatments and prognosis of the breast cancer patients [15–18], it is rational to hypothesize that rs2067474 might also be associated with breast cancer risk. However, as far as we know, no related investigations have been carried out.

Therefore, the goal of the present study was to demonstrate the relationship between rs2067474 of* HRH2* and breast cancer risk by using case-control method among Chinese Han population, hoping to provide further insights into the effect of HRH2 on the development of breast cancer.

## 2. Materials and Methods

### 2.1. Subjects

Two hundred and one Chinese Han women with breast cancer were recruited from our previous studies [17, 18], who were admitted to Kunming General Hospital of Chengdu Military Region between 2009 and 2014. Patients who had comorbidity, such as diabetes mellitus, hypertension, or any endocrine disorders, were excluded from the present study. We also recruited 238 unrelated, healthy Chinese Han women from the population of individuals referred for health examination in the same hospital as control group. All the participants in the control group had no known medical illness or hereditary disorders and were not taking any medications. Principal clinical characteristics, such as age at diagnosis, body mass index, and menopausal state, were obtained from the interviewer-administered health risk questionnaires. Menopausal status was defined as the date of last menses followed by 12 months of no menses. The clinicopathological variables and prognostic factors, that is, tumor size, histology, clinical stages, lymph node involvement, hormone receptor (including estrogen receptor and progesterone receptor) status, HER2 status, and p53 status, were obtained from the patients' medical records. In particular, The HER2-positive status of all tumors was confirmed with the use of fluorescence in situ hybridization. The criteria for hormone receptor positive indicate that the proportion of tumor cells immunostained was more than 10%, and if the proportion of tumor cells immunostained was less than 10%, then it is negative. The histologic determinations, including tumor type and disease stage, were performed according to the World Health Organization criteria and the TNM classification system, respectively.

The study protocol conformed to the ethical guidelines of the Declaration of Helsinki and was reviewed and approved by the Ethical Committee of Kunming General Hospital of Chengdu Military Region. Statement of informed consent was obtained from all participants after full explanation of the procedure.

### 2.2. Genotyping Assay

Sample preparation was as previously described [17, 18]. Briefly, the peripheral blood samples were collected into tubes containing ethylenediaminetetraacetic acid and stored at −80°C until analysis. Standard phenol–chloroform extraction method was used to extract genomic DNA from the whole blood. DNA concentration was measured by spectrometry (DU530 UV/VIS spectrophotometer, Beckman Instruments, Fullerton, CA, USA). Polymorphism of rs2067474 was genotyped by amplification-restriction and electrophoresis method as reported previously [14]. The primers used (according to the* HRH2* gene sequence Gene Bank Accession number AB023486) were as follows: forward 5′ACA GCC CGT GGC TAA GAA TGG3′ and reverse 5′AGA AGG GAG GCA GGA TGC AAG3′.

### 2.3. Statistical Analysis

Statistical analyses in the present study were performed with SPSS 18.0 for Windows (PASW Statistics, SPSS Inc., Chicago, IL). The level of significance was set at *P* < 0.05 (two-sided) for all tests. The SNP frequencies in both groups were tested for departure from Hardy–Weinberg Equilibrium (HWE). Student's *t*-test, analysis of variance (ANOVA), chi-square (Pearson's *χ* ^2^) test or Fisher's exact test, and unconditional multivariate logistic regression analysis adjusted for age, menopausal state, and body mass index (BMI) were used where necessary according to our previous reports [17, 18]. Odds ratios (ORs) with 95% confidential intervals (CIs) were used to assess the associations between genotypes and breast carcinoma risk or clinical variables.

## 3. Results

### 3.1. Participants' Characteristics

Descriptive clinical characteristics of both the breast cancer patients and health control subjects are listed in Table 1, which shows no significant differences in age and the distribution of menopausal state (*P* = 0.381 and 0.120, resp.) between the cases and controls. However, the BMI values of these two groups exhibit statistical difference (*P* = 0.049). As for breast cancer cases, majority of patients were diagnosed with clinical stage I or II (78.6%) or with ductal invasive carcinoma (87.6%). Furthermore, 58.0% and 72.1% of the patients were lymph node metastasis carriers and positive for hormone receptors, respectively. There were 98 patients (48.8%) who were HER-negative (0) or HER-weak positive (1) carriers.

### 3.2. Distributions of rs2067474 Genotype and Allele between Breast Cancer Patients and Health Controls

The overall genotype and allele frequencies of rs2067474 in breast cancer cases and health controls are shown in Table 2. The genotype distributions of rs2067474 in both groups were in the expected HWE (*P* = 0.152 for patients and 0.107 for controls, resp.). The minor allele frequency (MAF) of rs2067474 was 0.119 in case group and 0.166 in control group. We found no significant difference in the frequencies of genotypes (*P* = 0.174) or alleles (*P* = 0.054) between breast cancer patients and health controls according to univariate analysis (Table 2). Considering that variables of age, menopausal state, and BMI may affect the development of breast cancer, we further performed multivariate regression analysis according to 3 different genetic models (i.e., dominant, recessive, and additive genetic models). However, as shown in Table 3, we still did not observe any significant associations (*P* > 0.05) between rs2067474 polymorphism and breast cancer risk after adjustment for age, menopausal state, and BMI in all of the 3 genetic models.

### 3.3. Association between rs2067474 Genotype Distribution and Clinicopathological Parameters in Patients with Breast Cancer

Then, we further analyzed the genotype distribution of rs2067474 according to different clinicopathological parameters. As shown in Table 4, there were no statistical significances on the associations between genotype distribution of rs2067474 and nearly all selected clinicopathological parameters in the present study, except that the genotype distribution of rs2067474 was statistically correlated with the hormone receptor status of the present breast cancer patients (*P* = 0.016).

## 4. Discussion

Histamine has been known to play many important roles in proliferation, differentiation, and epithelial-to-mesenchymal transition of many kinds of tumors including breast cancer [19–23], whose effects are mediated by 4 different types of histamine receptors (i.e., from H_1_ to H_4_ receptors), respectively. As a result, the 4 types of histamine receptor genes might all be candidate genes associated with breast cancer. However, recent work has paid much attention to the relationships between breast cancer and two newly discovered histamine receptors (i.e., H_3_ and H_4_ receptors) [24, 25]. Our previous studies also focused on these two histamine receptors and found that polymorphism of H_4_ receptor gene, but not H_3_ receptor gene, was associated with the risk and malignant degree of breast cancer in Chinese Han populations [17, 18] while for HRH2 no study focused on the relationship between its gene polymorphism and breast cancer so far. Therefore, elucidating this issue might provide better understandings of how HRH2 affects the development of breast cancer.

Among* HRH2* gene polymorphisms, we selected one SNP, rs2067474, in the present study as this SNP was widely studied and reported to be associated with many kinds of diseases [2, 11–14]. Furthermore, this SNP is located in an enhancer element of* HRH2* gene promoter and is very likely to affect the transcription and expression of HRH2 [10, 13, 14]. According to the present data, the MAF of rs2067474 in control group (0.166) was close to the allele frequency data of the previous studies on East Asian populations [2] and also HapMap CHB data (http://www.hapmap.org/). Furthermore, the genotype distributions of rs2067474 in both case and control groups were in HWE, which demonstrates that the case-control samples consist of a representative population for the study. However, we did not observe any significant associations between rs2067474 and breast cancer risk in the present study.

It should be mentioned that, although HRH2 was found to be widely expressed in many other cancer cells and was suggested to be related to their proliferation and differentiation [12, 26, 27], early clinical investigations regarding its relationship with breast cancer did not reach a consistent conclusion [28, 29]. This may partially be due to the interactive hormone promoting effect of the currently available HRH2 antagonists [30–33], which was very likely to mask the eventual HRH2 blocking effect of these HRH2 antagonists. Therefore, since genetic association investigation does not involve the application of any HRH2 antagonists, our present findings may be more persuasive regarding the direct effect of HRH2 on breast cancer compared with previous investigations.

An interesting finding of the present study is that the genotype distribution of rs2067474 was statistically associated with the hormone receptor status. The underlying mechanisms are unknown yet. Some early investigations have indicated that histamine and HRH2 were involved in the regulation of pituitary hormone secretion [34, 35], which, in turn, might affect the expressions of estrogen receptor or progesterone receptors in breast tissue cells. Future work is needed to elucidate this possibility.

In summary, to our best knowledge, this work is the first study addressing the relationship between* HRH2* gene polymorphism and risk of breast cancer. However, no statistical association was observed regarding rs2067474 in this study, which indicates that this* HRH2* gene polymorphism might not be a risk factor in the development of breast cancer in Chinese Han population.

## Figures and Tables

**Table 1:** Characteristics of breast cancer patients and control participants.

	Breast cancer (*n* = 201)	Control (*n* = 238)	*P*
Age (years)	46.5 ± 9.2	47.2 ± 7.9	0.381^a^
BMI (kg/m^2^)	23.1 ± 2.9	22.6 ± 2.7	**0.049** ^a^
Sex			
Women	201 (100%)	238 (100%)	**—**
Menopausal state			
Premenopausal	126 (62.7%)	131 (55.0%)	0.120^b^
Postmenopausal	75 (37.3%)	107 (45.0%)	
Tumor size (cm)			
≤2.0	44 (21.9%)		
>2.0	157 (78.1%)		
Histology			
DIC	176 (87.6%)		
LIC	9 (4.4%)		
Others	16 (8.0%)		
Clinical stages			
I or II	158 (78.6%)		
III or IV	43 (21.4%)		
Lymph node metastasis			
Node-negative	116 (58.0%)		
Node-positive	85 (42.0%)		
Hormone receptor status			
Negative	56 (27.9%)		
Positive	145 (72.1%)		
HER2 status			
0-1	98 (48.8%)		
2-3	103 (51.2%)		

BMI: body mass index, DIC: ductal invasive carcinoma, and LIC: lobular invasive carcinoma.

^a^ *P* values were calculated by Student's *t*-tests.

^b^ *P* values were calculated from two-sided chi-square test.

**Table 2:** Frequency distributions of *HRH2* rs2067474 genotypes and allele and their associations with the risk of developing breast cancer.

rs2067474	Breast cancer	Control	*P* ^a^	OR (95% CI)
	*n* (%)	*n* (%)		
Genotype				
GG	158 (78.6)	169 (71.0)	0.174	—
GA	38 (18.9)	59 (24.8)		
AA	5 (2.5)	10 (4.2)		
Allele				
G	354 (88.1)	397 (83.4)	0.054	0.681 (0.463–1.003)
A	48 (11.9)	79 (16.6)		

OR: odd ratio, CI: confidence interval, and Ref: reference category.

^a^ *P* values were calculated from two-sided chi-square tests.

**Table 3:** Multivariate analysis for *HRH2* rs2067474 polymorphism and risk of breast cancer according to dominant, recessive, and additive genetic models.

	Dominant model	Recessive model	Additive model
	*P* ^a^; OR (95% CI)	*P* ^a^; OR (95% CI)	*P* ^a^; OR (95% CI)
rs2067474	0.065; 0.660 (0.424–1.027)	0.275; 0.543 (0.182–1.625)	0.056; 0.696 (0.480–1.010)

OR: odd ratio; CI: confidence interval.

^a^ *P* values were calculated by logistic regression adjusted for age, menopausal state, and body mass index.

**Table 4:** Correlations of clinicopathological parameters and *HRH2* rs2067474 polymorphism in patients with breast cancer.

	rs2067474			*P*
	GG	GA	AA	
Age (years)	46.4 ± 9.2	47.1 ± 8.8	45.6 ± 12.8	0.878^a^
BMI (kg/m^2^)				
≥25	33 (70.2%)	12 (25.5%)	2 (4.3%)	0.206^b^
<25	125 (81.2%)	26 (16.9%)	3 (1.9%)	
Menopausal state				
Premenopausal	102 (81.0%)	21 (16.7%)	3 (2.4%)	0.558^b^
Postmenopausal	56 (74.7%)	17 (22.7%)	2 (2.7%)	
Tumor size (cm)				
≤2.0	37 (84.1%)	5 (11.4%)	2 (4.5%)	0.203^b^
>2.0	121 (77.1%)	33 (21.0%)	3 (1.9%)	
Histology				
DIC	137 (77.8%)	36 (20.5%)	3 (1.7%)	0.116^b^
LIC	8 (88.9%)	1 (11.1%)	0 (0.0%)	
Others	13 (81.3%)	1 (6.3%)	2 (12.5%)	
Clinical stages				
I or II	124 (78.5%)	30 (19.0%)	4 (2.5%)	1.000^b^
III or IV	34 (79.1%)	8 (18.6%)	1 (2.3%)	
Lymph node metastasis				
Node-negative	89 (76.7%)	23 (19.8%)	4 (3.4%)	0.606^b^
Node-positive	69 (81.2%)	15 (17.6%)	1 (1.2%)	
Hormone receptor status				
Negative	45 (80.4%)	7 (12.5%)	4 (7.1%)	**0.016** ^b^
Positive	113 (77.9%)	31 (21.4%)	1 (0.7%)	
HER2 status				
0-1	81 (82.7%)	16 (16.3%)	1 (1.0%)	0.267^b^
2-3	77 (74.8%)	22 (21.4%)	4 (3.9%)	
p53 status				
Negative	41 (78.8%)	9 (17.3%)	2 (3.8%)	0.311^b^
Positive	62 (77.5%)	18 (22.5%)	0 (0.0%)	
Undetermined	55 (79.7%)	11 (15.9%)	3 (4.3%)	

OR: odd ratio, CI: confidence interval, BMI: body mass index, DIC: ductal invasive carcinoma, LIC: lobular invasive carcinoma, *HER2*: human epidermal growth factor receptor, and p53: tumor protein 53.

^a^ *P* values were calculated by analysis of variance (ANOVA).

^b^ *P* values were calculated from two-sided chi-square tests or Fisher's exact tests.

## Abbreviations

BMI: Body mass index
CI: Confidence interval
DIC: Ductal invasive carcinoma
HRH2: Histamine H_2_ receptor
HWE: Hardy-Weinberg Equilibrium
LIC: Lobular invasive carcinoma
ORs: Odds ratios.

## References

[1] Traiffort E., Ruat M., Arrang J.-M., Leurs R., Piomelli D., Schwartz J.-C. (1992). Expression of a cloned rat histamine H2 receptor mediating inhibition of arachidonate release and activation of cAMP accumulation. *Proceedings of the National Academy of Sciences of the United States of America*.

[2] Yamada H., Tahara T., Shiroeda H. (2012). Effects of -1018G>A polymorphism of HRH2 (rs2607474) on the severity of gastric mucosal atrophy. *Journal of Gastrointestinal and Liver Diseases*.

[3] He G., Hu J., Li T. (2012). Arrhythmogenic effect of sympathetic histamine in mouse hearts subjected to acute ischemia. *Molecular Medicine*.

[4] Haas H. L., Sergeeva O. A., Selbach O. (2008). Histamine in the nervous system. *Physiological Reviews*.

[5] Davio C., Baldi A., Mladovan A. (1995). Expression of histamine receptors in different cell lines derived from mammary gland and human breast carcinomas. *Inflammation Research*.

[6] Cricco G. P., Davio C. A., Martin G. (1994). Histamine as an autocrine growth factor in experimental mammary carcinomas. *Agents and Actions*.

[7] Davio C. A., Cricco G. P., Martin G., Fitzsimons C. P., Bergoc R. M., Rivera E. S. (1994). Effect of histamine on growth and differentiation of the rat mammary gland. *Agents and Actions*.

[8] Parshad R., Hazrah P., Kumar S., Gupta S. D., Ray R., Bal S. (2005). Effect of preoperative short course famotidine on TILs and survival in breast cancer. *Indian Journal of Cancer*.

[9] Porretti J. C., Mohamad N. A., Martín G. A., Cricco G. P. (2014). Fibroblasts induce epithelial to mesenchymal transition in breast tumor cells which is prevented by fibroblasts treatment with histamine in high concentration. *International Journal of Biochemistry and Cell Biology*.

[10] García-Martín E., Ayuso P., Martínez C., Blanca M., Agúndez J. A. G. (2009). Histamine pharmacogenomics. *Pharmacogenomics*.

[11] Mancama D., Arranz M. J., Munro J. (2002). Investigation of promoter variants of the histamine 1 and 2 receptors in schizophrenia and clozapine response. *Neuroscience Letters*.

[12] Arisawa T., Tahara T., Ozaki K. (2012). Association between common genetic variant of HRH2 and gastric cancer risk. *International Journal of Oncology*.

[13] Nomura T., Tahara T., Shiroeda H. (2013). Influence of HRH2 promoter polymorphism on aberrant DNA methylation of DAPK and CDH1 in the gastric epithelium. *BMC Gastroenterology*.

[14] García-Martín E., Ayuso P., Luengo A., Martínez C., Agúndez J. A. G. (2008). Genetic variability of histamine receptors in patients with Parkinson's disease. *BMC Medical Genetics*.

[15] González-Neira A. (2012). Pharmacogenetics of chemotherapy efficacy in breast cancer. *Pharmacogenomics*.

[16] Justenhoven C., Obazee O., Brauch H. (2012). The pharmacogenomics of sex hormone metabolism: breast cancer risk in menopausal hormone therapy. *Pharmacogenomics*.

[17] He G.-H., Lu J., Shi P.-P. (2013). Polymorphisms of human histamine receptor H4 gene are associated with breast cancer in Chinese Han population. *Gene*.

[18] He G.-H., Lin J.-J., Cai W.-K. (2014). Associations of polymorphisms in histidine decarboxylase, histamine N-methyltransferase and histamine receptor H_3_ genes with breast cancer. *PLoS ONE*.

[19] Medina V., Cricco G., Nuñez M. (2006). Histamine-mediated signaling processes in human malignant mammary cells. *Cancer Biology and Therapy*.

[20] Medina V. A., Rivera E. S. (2010). Histamine receptors and cancer pharmacology. *British Journal of Pharmacology*.

[21] Blaya B., Nicolau-Galmés F., Jangi S. M. (2010). Histamine and histamine receptor antagonists in cancer biology. *Inflammation and Allergy - Drug Targets*.

[22] Francis H., DeMorrow S., Venter J. (2012). Inhibition of histidine decarboxylase ablates the autocrine tumorigenic effects of histamine in human cholangiocarcinoma. *Gut*.

[23] Cai W.-K., Hu J., Li T. (2014). Activation of histamine H4 receptors decreases epithelial-to-mesenchymal transition progress by inhibiting transforming growth factor-*β*1 signalling pathway in non-small cell lung cancer. *European Journal of Cancer*.

[24] Medina V., Croci M., Crescenti E. (2008). The role of histamine in human mammary carcinogenesis: H3 and H4 receptors as potential therapeutic targets for breast cancer treatment. *Cancer Biology and Therapy*.

[25] Medina V. A., Brenzoni P. G., Lamas D. J. M. (2011). Role of histamine H4 receptor in breast cancer cell proliferation. *Frontiers in Bioscience*.

[26] Aurelius J., Martner A., Brune M. (2012). Remission maintenance in acute myeloid leukemia: impact of functional histamine H2 receptors expressed by leukemic cells. *Haematologica*.

[27] Pagotto R. M., Monzón C., Moreno M. B., Pignataro O. P., Mondillo C. (2012). Proliferative effect of histamine on MA-10 leydig tumor cells mediated through HRH2 activation, transient elevation in cAMP production, and increased extracellular signal-regulated kinase phosphorylation levels. *Biology of Reproduction*.

[28] Bowrey P. F., King J., Magarey C. (2000). Histamine, mast cells and tumour cell proliferation in breast cancer: does preoperative cimetidine administration have an effect?. *British Journal of Cancer*.

[29] Bolton E., King J., Morris D. L. (2000). H2-antagonists in the treatment of colon and breast cancer. *Seminars in Cancer Biology*.

[30] Michnovicz J. J., Galbraith R. A. (1991). Cimetidine inhibits catechol estrogen metabolism in women. *Metabolism*.

[31] Tworoger S. S., Eliassen A. H., Rosner B., Sluss P., Hankinson S. E. (2004). Plasma prolactin concentrations and risk of postmenopausal breast cancer. *Cancer Research*.

[32] Coogan P. F., Zhang Y., Palmer J. R., Strom B. L., Rosenberg L. (2005). Cimetidine and other histamine_2_-receptor antagonist use in relation to risk of breast cancer. *Cancer Epidemiology Biomarkers and Prevention*.

[33] Mathes R. W., Malone K. E., Daling J. R., Porter P. L., Li C. I. (2008). Relationship between histamine2-receptor antagonist medications and risk of invasive breast cancer. *Cancer Epidemiology Biomarkers and Prevention*.

[34] Moguilevsky J. A., Szwarcfarb B., Faigon M. R., Paolini J., Scacchi P. (1989). Effects of H1 and H2 histamine receptor antagonists on positive feed-back effect of estrogen on LH in prepubertal female rats. *Hormone and Metabolic Research*.

[35] Knigge U., Warberg J. (1991). The role of histamine in the neuroendocrine regulation of pituitary hormone secretion. *Acta Endocrinologica*.

